# Gut microbiota interacting with vitamin D but not anandamide might contribute to the pathogenesis of preeclampsia: a preliminary study

**DOI:** 10.3389/fcimb.2024.1469054

**Published:** 2025-02-05

**Authors:** Xiao-Qiang Han, Hui-Hui Jiang, Meng-Ling Chen, De-Yang Han, Su-Fen Zhou, Jin-Wen Wang, Shu-Shen Ji, Ling-Yun Wang, Jing-Wei Lou, Ming-Qun Li

**Affiliations:** ^1^ Department of Obstetrics and Gynaecology, Xiangyang No. 1 People’s Hospital, Hubei University of Medicine, Xiangyang, China; ^2^ Hubei Provincial Clinical Research Center for Accurate Fetus Malformation Diagnosis, Xiangyang, China; ^3^ Zhangjiang Center for Translational Medicine, Shanghai Biotecan Pharmaceuticals Co., Ltd., Shanghai, China; ^4^ Department of Ultrasound, Xiangyang No. 1 People’s Hospital, Hubei University of Medicine, Xiangyang, China

**Keywords:** gut microbiota, anandamide, vitamin D, preeclampsia, inflammation, interaction

## Abstract

**Introduction:**

Preeclampsia (PE) is a pregnancy-specific multisystem disorder and a leading cause of maternal and perinatal mortality globally. Despite numerous studies highlighting the potential roles of gut microbiota, anandamide (AEA), and Vitamin D (VitD) in PE, none have established them as reliable biomarkers for predicting disease onset. Moreover, their interactions in late-stage pregnancy women remain poorly understood.

**Methods:**

Thirty-four preeclamptic patients (called PE group) and thirty-nine matched healthy late-pregnant women (called LP group) were involved in this case-control study. Fecal samples, which were used to acquire the diversity and composition of gut microbiota, were analyzed by 16S rRNA gene sequencing. Plasma AEA concentrations and serum VitD levels were determined by high-performance liquid chromatography-mass spectrometry (HPLC-MS) and liquid chromatography-tandem mass spectrometry (LC-MS/MS), respectively.

**Results:**

In this study, β diversity but not α diversity significantly differed between the LP and PE groups. Compared with the LP group, the relative abundances of *Prevotella*, *Erysipelotrichaceae_UCG-003*, and *Dorea* were increased dramatically in the PE group, whereas the relative abundances of *Subdoligranulum*, *Parabacteroides*, *Bacteroides* were significantly decreased in the PE group. Furthermore, women with PE had a substantially lower plasma level of AEA and a marked decrease in serum VitD compared to normal late-pregnant women. Lastly, although the serum level of AEA was not significantly correlated with VitD or any of the top 6 marker genera, VitD was significantly negatively correlated with the relative abundance of *Dorea*, a novel finding in this context.

**Discussion:**

The gut microbiota profile of the PE group was significantly different from that of the LP group. Although no significant correlations were identified between the plasma AEA levels and serum VitD levels or any of the top 6 identified marker genera, a significant negative correlation was observed between VitD and Dorea, indicating VitD and gut microbiota have the potential to be combined targets for early diagnosis and management of PE.

## Introduction

As a leading cause of maternal and neonatal mortality, preeclampsia (PE) is a complex pregnancy disorder, that affects 3%-8% of pregnancies globally (Abalos et al., 2013; American College of Obstetricians and Gynecologists, 2020). PE not only elevates the risk of adverse pregnancy outcomes (e.g. preterm birth and low birth weight) (Rana et al., 2019) but is also linked to severe maternal and child health issues, such as end-stage kidney disease for mothers (Phipps et al., 2019; Turbeville and Sasser, 2020) and bronchopulmonary dysplasia for offspring (Ratsep et al., 2016; Phipps et al., 2019). Despite numerous studies on PE in recent years, there remain various etiological theories, including placental dysfunction, immune system maladaptation, oxidative stress, angiogenic imbalance, genetic predisposition, and nutritional deficiency (Udenze, 2016; Jabalie et al., 2019). The exact pathogenesis of PE is still not completely understood, and termination of the pregnancy remains the only definitive cure. Therefore, a multi-dimensional investigation into the mechanisms of PE could reveal new potential targets for its early diagnosis and management, which might be crucial in preventing its onset and improving its prognosis.

The gut microbiota, a complex and vast community of microorganism species residing in the digestive tract, plays a crucial role in host metabolism, immunity, and nutrient absorption (Viennois and Chassaing, 2018). Emerging research suggests that the dysbiosis of gut microbiota may be involved in the development of PE. The altered composition of gut bacteria could promote inflammation, metabolic changes, and immune dysregulation, all of which are linked to PE. Compared to healthy controls, significant alterations of gut microbiota and their metabolites were observed in patients with PE (Gomez-Arango et al., 2016; Liu et al., 2017; Lv et al., 2019; Wang et al., 2019). Recent studies have indicated that short-chain fatty acids (SCFAs) are involved in regulating the levels of blood pressure in both patients with PE and PE pregnant rats (Chang et al., 2020; Yong et al., 2022). Moreover, the dysbiosis of gut microbiome disrupted the gut barrier, leading to the colonization of intestinal bacteria in the uterine cavity, thus causing PE (Chen et al., 2020). However, the findings from published studies are inconsistent. Although multiple studies have found that *Bifidobacterium* has a protective effect against PE (Ahmadian et al., 2020; Chen et al., 2020; Altemani et al., 2021), Altemani et al. observed an increased abundance of *Bifidobacterium* in patients with PE (Altemani et al., 2021). Miao and Lv et al. identified *Blautia* as a risk factor for PE (Lv et al., 2019; Miao et al., 2021), whereas Chang and Yu reported the opposite (Chang et al., 2020; Yu et al., 2022). Therefore, it is essential to continue exploring the characteristics of gut microbiome in patients with PE by expanding the sample size and geographical scope.

Except for the dysbiosis of gut microbiota, alterations in the expression of the endocannabinoid system (ECS) are also linked to the development of PE. Compared with healthy pregnant women, women with PE exhibited reduced levels of anandamide (AEA) in both plasma and placental tissues (Molvarec et al., 2015; Maia et al., 2023). Meanwhile, decreased levels of other ECS components, such as N-oleoylethanolamine (OEA) and N-docosahexaenoylethanolamine (DHEA), were also observed in placental tissues (Maia et al., 2023). Interestingly, both ESC-G-protein coupled receptors 1 and 2 (CB1 and CB2) were expressed on enteric nerves, enterocytes, and immune cells in the gut, and the balance of ECS was affected by the gut microbiota. Moreover, the unbalance of ECS could in turn impact the integrity of the intestinal barrier (Moludi et al., 2018). For instance, when CB1 receptor antagonist SR141716A was administered orally to mice with diet-induced obesity (DIO), it led to attenuation in inflammatory cytokines of adipose tissue, changes in gut microbiome composition, and enhancement of mucus layer thickness, compared to DIO mice receiving a vehicle (Mehrpouya-Bahrami et al., 2017). In addition, the administration of *Lactobacillus casei* in antibiotic-treated mice not only alleviated depressive-like behaviors but also restored the concentrations of N-acyl-serotonin, which in turn normalized the concentrations of AEA in the gastrointestinal tract (Guida et al., 2018). Inspired by the interactions between gut microbiota and ECS components in animal models of human disease, we aimed to explore the associations between gut microbiota and AEA in patients with PE.

Nutritional deficiencies, such as low levels of vitamin D (VitD), also participated in the occurrence and progression of PE. VitD deficiency could impair placental development, angiogenesis, and immune system. Except for the closed linkage between VitD deficiency and cardiovascular diseases or arterial hypertension in observational studies (Pilz et al., 2009; Wimalawansa, 2018), both observational studies (Achkar et al., 2015; Baca et al., 2016; Serrano et al., 2018) and meta-analyses (Wei et al., 2013; Akbari et al., 2018; Serrano-Diaz et al., 2018; Aguilar-Cordero et al., 2020; Hu et al., 2022) have reported a significant association between VitD deficiency and an increased risk of PE. According to the current evidence, the associations between VitD levels and gut microbial structure and function are sufficient (Ooi et al., 2013; Jin et al., 2015; Waterhouse et al., 2019). The maternal gut microbiome is also associated with the intake of dietary VitD by mothers (Mandal et al., 2016). However, it is still unknown whether there are interactions among gut microbial compositions, AEA levels, and VitD levels in late-pregnant women with or without PE. On these bases, we aimed to provide new insights into the interactions of these risk factors by examining the structure and composition of gut microbiota, the plasma levels of AEA, and the serum levels of VitD, which may systematically provide evidence of multi-dimensional pathogenesis and intervention strategies of PE.

## Methods

### Ethics statement

This study received approval from the Ethical Committee of Biomedical Basic Research of Xiangyang No. 1 People’s Hospital, Hubei University of Medicine (Xiangyang, China). Its corresponding Institutional Review Board (IRB) number was XYYYE20220052. Written consent was obtained from all participants for using their data and samples prior to enrollment by the Declaration of Helsinki.

### Patients and groups

This case-control study included 34 preeclamptic patients (called PE group) and 39 normal late-pregnant women (called LP group) with uncomplicated pregnancies. Maternal characteristics such as age, height, weight, and gestational age were collected. The pregnancy body mass index (BMI) was calculated by dividing the weight (kg) by the square of the height (meters). Inclusion criteria for the PE group followed the diagnostic standards of the American College of Obstetricians and Gynecologists (James et al., 2013), which include blood pressure ≥140/90mmHg for two consecutive readings at least 4 hours apart, and proteinuria ≥300mg, or in the absence of proteinuria, any of the following: thrombocytopenia, renal insufficiency, impaired liver function, pulmonary edema, or cerebral or visual symptoms. The exclusion criteria for this recruitment were: (1) subjects were not local residents; (2) multiple pregnancies; (3) pre-existing chronic diseases (e.g. diabetes, hypertension, inflammatory bowel disease, chronic kidney disease), autoimmune disorders (e.g., systemic lupus erythematosus, rheumatoid arthritis, sjögren’s syndrome, scleroderma, mixed connective tissue disease, hashimoto’s thyroiditis, graves’ disease, multiple sclerosis, guillain-barré syndrome, myasthenia gravis, psoriasis, vitiligo, pemphigus and pemphigoid, autoimmune hemolytic anemia, immune thrombocytopenic purpura, antiphospholipid syndrome, goodpasture’s syndrome, IgA nephropathy), liver diseases (e.g., hepatitis, cirrhosis), malabsorption syndromes (e.g., celiac disease, bariatric surgery), malignant tumors, depression, or other complications before pregnancy; (4) use of medication and supplement (e.g. antibiotics, probiotics, prebiotics, chronic steroid, immunosuppressant, vitamin D) within 3 months prior to the study; (5) follow strict diets (e.g., veganism, ketogenic diets), malnutrition, extreme dietary habits; (6) use of tobacco, alcohol, illicit drugs, substance abuse; (7) allergies to soy, probiotics or prebiotics; (8) lactose intolerance; (9) family history of preeclampsia or eclampsia; (10) previous history of preeclampsia or eclampsia; (11) maternal or fetal infection and fetal congenital anomalies.

## Collection of fecal samples

Fecal specimens (approximately the size of two soybean grains) were acquired from each subject either by themselves or their family members at home or in the hospital within 3 minutes of defecation. After collection, the fecal samplers (Biotecan, Shanghai, China) were sealed, labeled, and transferred to Biotecan Laboratories at temperatures below 18°C within 2 days. Upon arrival, they were stored at -80°C.

### 16S rRNA gene sequencing and data processing

A total of 73 fecal samples (LP vs. PE = 39 vs. 34) were collected in fecal samplers and stored at -80°C until used for high-throughput sequencing. Bacterial genomic DNA was extracted using the QIAamp PowerFecal Pro DNA Kit (QIAGEN, Germany). The extracted DNA was then amplified using the Phusion High-Fidelity PCR Master Mix (New England Biolabs, Massachusetts, USA), targeting the V3V4 region of the 16S rRNA genes with the forward primer 341F (5′-CCTACGGGNGGCWGCAG-3′) and the reverse primer 805R (5′-GACTACHVGGGTATCTAATCC-3′). The PCR products were purified using the TransStart^®^ FastPfu DNA Polymerase kit (TransGen, Beijing, China). The purified DNA was quantified using the Qubit dsDNA HS Assay Kit (Thermo Fisher Scientific, Massachusetts, USA). Library quantification was performed with the Library Quant Kit Illumina GA revised primer-SYBR Fast Universal (KAPA Biosystems, Massachusetts, USA), and a Novaseq6000 500 cycle (Illumina, California, USA) was adopted to perform pair-end 2 × 250bp sequencing.

To analyze these sequencing data, the Quantitative Insights Into Microbial Ecology 2 (QIIME 2, v2017.6.0) pipeline and established criteria were employed (Gill et al., 2006; Caporaso et al., 2010). Vsearch V2.4.4 was utilized to assemble the paired-end reads (Rognes et al., 2016). Operational taxonomic units (OTUs) were assigned based on 16S rRNA gene sequences with a similarity cutoff of 97%, referencing the Greengenes database via Vsearch V2.4.4. Notably, OTUs representing less than 0.001% of the total sequences were excluded. The final OTU table was generated by averaging, rounding, and rarefying, based on 100 evenly resampled OTU subsets at 90% of the minimum sequencing depth. Abundance curves were plotted at the OTU level, and sequencing depth was assessed and confirmed through rarefaction analysis.

### Bioinformatics and statistical analyses

The chi-square test was adopted to assess statistical differences in categorical variables between the LP and PE groups by SPSS 23.0 (IBM, Chicago, IL, USA). Continuous variables were expressed as medians with interquartile ranges (IQR) and compared between groups using the Mann–Whitney U-test in GraphPad Prism version 7.0 (GraphPad, San Diego, CA, USA). Venn diagrams, heat maps, and correlation analyses were conducted with R software (v3.6.3). The phylogenetic tree was illustrated using GraPhlAn (http://huttenhower.sph.harvard.edu/GraPhlAn).

Alpha diversity analysis (Chao1 index, Simpson index, and Shannon index) was conducted using QIIME 2. Statistical comparisons were performed using the Pairwise Wilcox test. Beta diversity analysis was performed by Weighted UniFrac principal component analysis (PCoA). The composition and structure of intestinal bacteria between the LP and PE groups were compared by Permutational multivariate analysis of variance (PERMANOVA). One-way analysis of similarities (Anosim) was used to assess the comparability between groups. Linear discriminant analysis effect size (LEfSe) was employed to identify taxa with significantly different abundances across groups based on default parameters (logarithmic LDA score = 2) (Segata et al., 2011). The resulting OTU table was analyzed using BugBase (http://github.com/danknights/bugbase) to assess microbial phenotype differences between the LP and PE groups (Thomas et al., 2016). Phylogenetic investigation of communities by reconstruction of unobserved states (PICRUSt, PICRUSt2 v2.3.0-b) was utilized to predict gut microbial functions (Langille et al., 2013), and the Univariate Test was adopted to assess the significant difference.

## Collection of maternal peripheral blood samples

Maternal blood samples were obtained from an antecubital vein into plain tubes with anticoagulant for AEA detection and plain tubes without additives for VitD detection. These blood samples were centrifuged at room temperature with a relative centrifugal force of 4000 rpm for 10 min. The centrifuged plasma and serum were stored at -80°C until further analysis.

## Detection of plasma AEA

Plasma concentrations of AEA were quantified using high-performance liquid chromatography-mass spectrometry (HPLC-MS) methodology. Firstly, 50 μl plasma samples were mixed with 200 μl internal standard solution (100 ng/ml Arachidonoyl Ethanolamide-d8, Cayman Chemical, Art. No. 390050-500). The samples were vortexed for 5 minutes at 1400 rpm and centrifuged at 4000 rpm for 10 minutes. Following centrifugation, 120 μl of the supernatant was extracted for analysis. Quantification was performed using a calibration curve established from blank plasma samples spiked with varying concentrations of AEA, ranging from 0.125 to 4.000 ng/ml. The HPLC system was AB SCIEX Triple Quad™ 4500MD (SCIEX, Boston, USA) with an electrospray ionization source operated in positive ion mode. Separation of analytes was achieved through reversed-phase liquid chromatography using a Waters BEH-C18 column (Waters Corporation, Delaware, United States) with gradient elution. Mobile phase A consisted of 0.1% formic acid solution and mobile phase B was composed of 0.1% formic acid in methanol. The flow rate of the mobile phases was maintained at 450 μl/min during analysis.

## Detection of serum VitD

Serum levels of 25(OH)D2 and 25(OH)D3 were quantified using the liquid chromatography tandem mass spectrometry (LC-MS/MS) method. Firstly, 50 μl of serum samples were combined with 200 μl of isotope-labeled internal standard solution (20 ng/ml 25(OH)D3-d6, Sigma, Art. No. H-074). Secondly, the samples were vortexed at 1400 rpm for 5 minutes and centrifuged at 4000 rpm for 10 minutes. Thirdly, 100 μl supernatant was transferred into a 96-deep well plate and dried under nitrogen at 45°C. Fourthly, derivatization was performed using a 60 μl acetonitrile solution containing 1 μg/ml PTAD for 5 minutes, and subsequently added 60 μl water for reconstitution. Fifthly, the prepared samples were injected into the UPLC-MS/MS system (Waters Corporation, Delaware, United States). Chromatographic separation was achieved on the Waters ACQUITY UPLC I-class using a binary gradient mobile phase consisting of water with 5 mM methylamine (mobile phase A) and methanol with 5 mM methylamine (mobile phase B). The flow rate of the mobile phases was set at 400 μl/min, and the column temperature was maintained at 40°C. MS/MS detection was conducted using the Xevo TQD in positive electrospray ionization mode with multiple reaction monitoring (MRM) mode.

## Results

### Differences in demographic characteristics between the LP group and the PE group

**Table 1** summarizes the baseline characteristics of both the LP and PE groups. No significant differences were observed in terms of age, BMI, or gestational age between these two groups.

**Table 1:** Demographic characteristics of healthy pregnant women and preeclamptic patients.

Groups	Healthy late-pregnant women	Preeclamptic patients	*P* value
Subjects, n	39	34	
Age (years)	30.26 (27.17–33.09)	29.50 (26.76–32.52)	NS
Gestational weeks (weeks)	39.00 (38.29–39.43)	38.43 (37.57–39.36)	NS
BMI (kg/m2)	26.29 (25.24–28.34)	27.49 (25.49–30.45)	NS
Number of pregnancies	2 (1–3)	1 (1–2)	<0.05
Production times	1 (0–1)	0 (0–0)	<0.001
Education Level			
Primary school	1	0	NS
Junior high school	1	1	
Senior high school	6	6	
Junior college	22	13	
Undergraduate	9	13	
Master	0	1	
Eat cured meat			
Yes	7	4	NS
No	32	30	
Antiabortifacient drugs			
Yes	11	6	NS
No	28	28	
Systolic blood pressure (mm Hg)	120.0 (112.0–128.0)	145.5 (143.0–147.3)	<0.0001
Diastolic blood pressure (mm Hg)	72.00 (65.00–78.00)	93.50 (91.00–96.00)	<0.0001
Fetal birth weight (g)	3300 (3150–3550)	3150 (2888–3575)	NS

BMI, body mass index; NS, not significant.Data are presented as median (interquartile range) for continuous variables and as number (percentage) for categorical variables.

### Differences in structure and composition of gut microbiota between the LP group and the PE group

**Figure 1:**
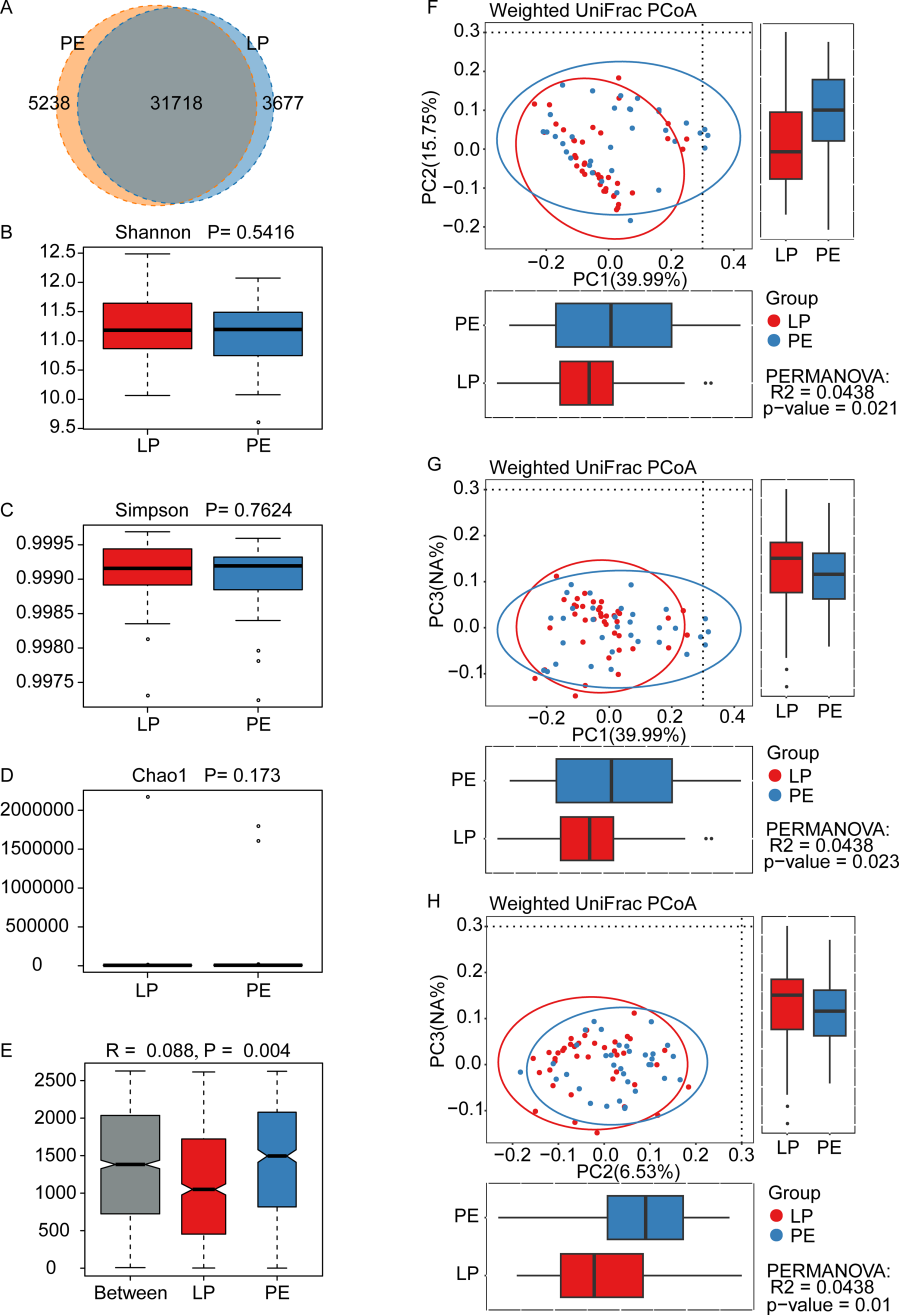
Diversity of gut microbiota in healthy late-pregnant women (LP, n=39) and preeclamptic patients (PE, n=34). **(A)** 31718 OTUs were shared between these two groups. The LP group had the unique OTUs with 3677, while the PE group had 5238. The indexes of Shannon **(B)**, Simpson **(C)**, and Chao1 **(D)** were adopted to evaluate alpha diversity (meaning the gut microbial richness and evenness) between the LP group and the PE group, and were obtained by Wilcoxon Rank Sum Test. All these three indexes did not show any statistically significant differences, whose P-values were 0.5416 for Shannon, 0.7624 for Simpson, and 0.173 for Chao1. **(E)** Analysis of similarity (ANOSIM) indicated the differences between the LP group and the PE group (p=0.004) were significantly greater than the differences within the groups, and our grouping was meaningful. **(F-H)** The beta diversity between the LP group and the PE group was analyzed *by* the Weighted UniFrac principal component analysis, and significant differences were observed between PC1 and PC2 (p=0.021) **(F)**, between PC1 and PC3 (p=0.023) **(G)**, and between PC2 and PC3 (p=0.01) **(H)**.

In the Venn diagram, there were 31,718 shared OTUs between the LP and PE groups, with the PE group exhibiting more unique OTUs (5,238) compared to the LP group (3,677) (**Figure 1A**); NCBI BioProject database PRJNA1137103. Regarding α-diversity, there were no statistically significant differences in gut microbial richness and evenness between these two groups (Shannon P=0.5416, Simpson P=0.7624, and Chao1 P=0.173) (**Figures 1B–D**). In terms of β-diversity, differences between groups were more pronounced than within the group (R=0.088, P<0.01) (**Figure 1E**). Weighted UniFrac principal component analysis (PCoA) indicated significant differences in the composition of gut microbiota between the LP group and the PE group (p<0.05) (**Figures 1F–H**).

### Differences in marker genera between the LP group and the PE group

**Figure 2:**
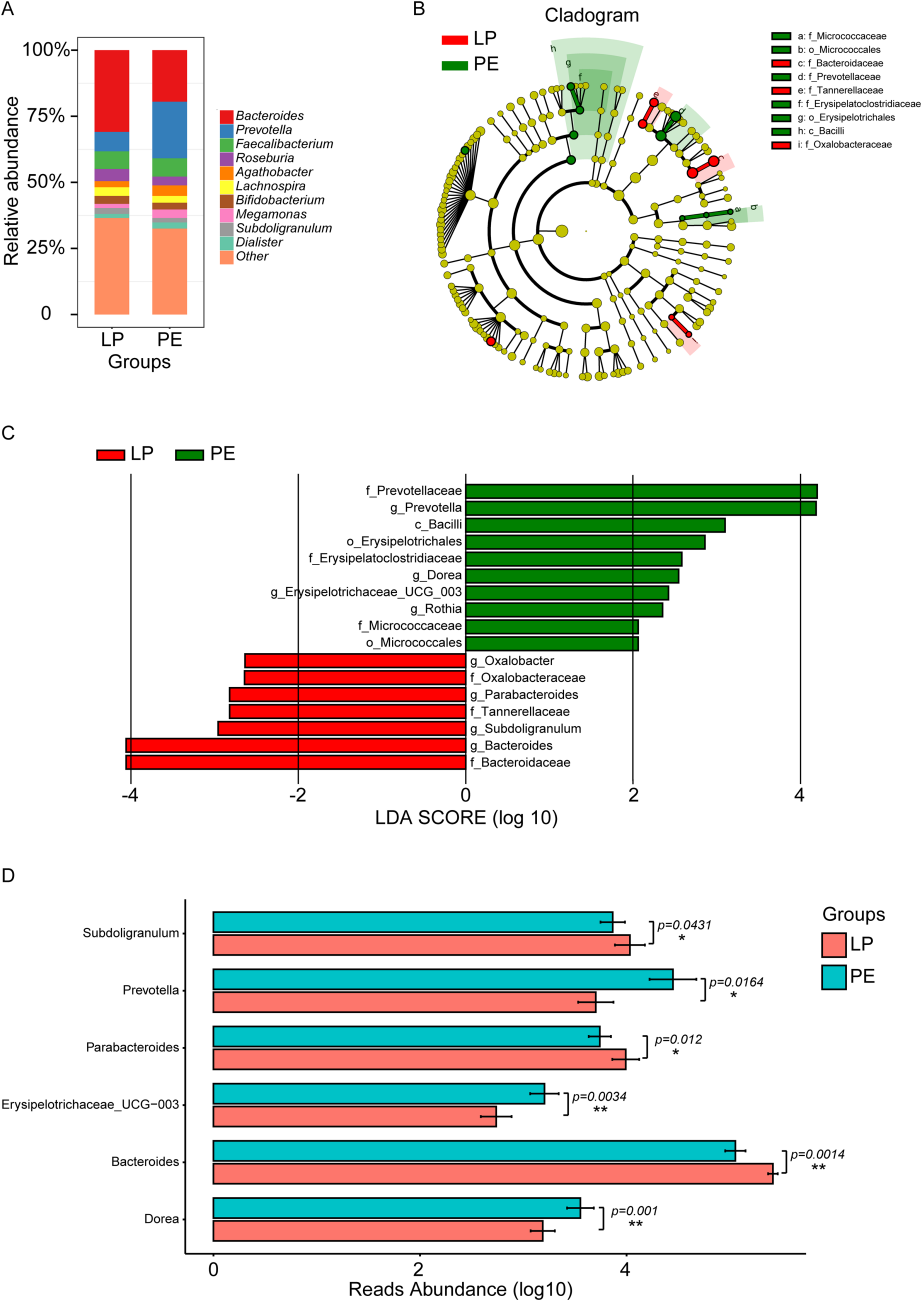
Composition and marker genera of gut microbiota in the LP and PE groups. **(A)** The relative abundance histograms of all genera in these two groups. The top 10 shared genera with high relative abundance were represented by different colors, including *Bacteroides*, *Prevotella*, *Faecalibacterium*, *Roseburia*, *Agathobacter*, *Lachnospira*, *Bifidobacterium*, *Megamonas*, *Subdoligranulum*, and *Dialister*, while the remaining genera with lower relative abundance were grouped as ‘other.’ **(B)** The phylogenetic tree illustrated the marker taxa based on the hierarchical relationship from phylum to species levels for these two groups. **(C)** The Wilcoxon rank sum test (LEfSe) (P<0.05, LDA>2) identified marker genera distinguishing between the LP group and the PE group. *Prevotella*, *Dorea*, *Erysipelotrichaceae_UCG-003*, and *Rothia* were identified as marker genera for the PE group, while *Bacteroides*, *Subdoligranulum*, *Parabacteroides*, and *Oxalobacter* were markers for the LP group. **(D)** Significant differences in genera between these two groups were acquired by the Univariate Test, including *Subdoligranulum* (p=0.0431), *Prevotella* (p=0.0164), *Parabacteroides* (p=0.0120), *Erysipelotrichaceae_UCG-003* (p=0.0034), *Bacteroides* (p=0.0014), and *Dorea* (p=0.0010). *p<0.05, **p<0.01.

Our focus primarily centered on downstream analysis at a genus level because of the restriction of 16S rDNA amplicon pyrosequencing. **Figure 2A** and **Supplementary Figure 1** depicted the gut microbial composition at this level. *Bacteroides*, *Prevotella*, *Faecalibacterium*, *Roseburia*, *Agathobacter*, *Lachnospira*, *Bifidobacterium*, *Megamonas*, *Subdoligranulum*, and *Dialister* emerged as prominent components in both the LP and PE groups. However, *Prevotella*, *Faecalibacterium*, *Agathobacter*, *Megamonas*, and *Dialister* exhibited higher prevalence in the PE group, whereas *Bacteroides*, *Roseburia*, *Lachnospira*, *Bifidobacterium*, and *Subdoligranulum* were more abundant in the LP group. The Wilcoxon rank sum test (LEfSe) (P<0.05, LDA>2) identified marker genera distinguishing between the groups, with the PE group featuring a greater number of marker bacteria compared to the LP group (**Figures 2B, C**). *Prevotella*, *Dorea*, *Erysipelotrichaceae_UCG-003*, and *Rothia* were identified as marker genera for the PE group, while *Bacteroides*, *Subdoligranulum*, *Parabacteroides*, and *Oxalobacter* were markers for the LP group (**Figure 2C**). Furthermore, we determined statistical significance for the top 6 differential genera, with P values of 0.0010, 0.0014, 0.0034, 0.0120, 0.0164, and 0.0431 for *Dorea*, *Bacteroides*, *Erysipelotrichaceae_UCG-003*, *Parabacteroides*, *Prevotella*, and *Subdoligranulum*, respectively (**Figure 2D**).

### Differences in phenotypic characteristics and potential metabolic pathways between the LP group and the PE group

**Figure 3:**
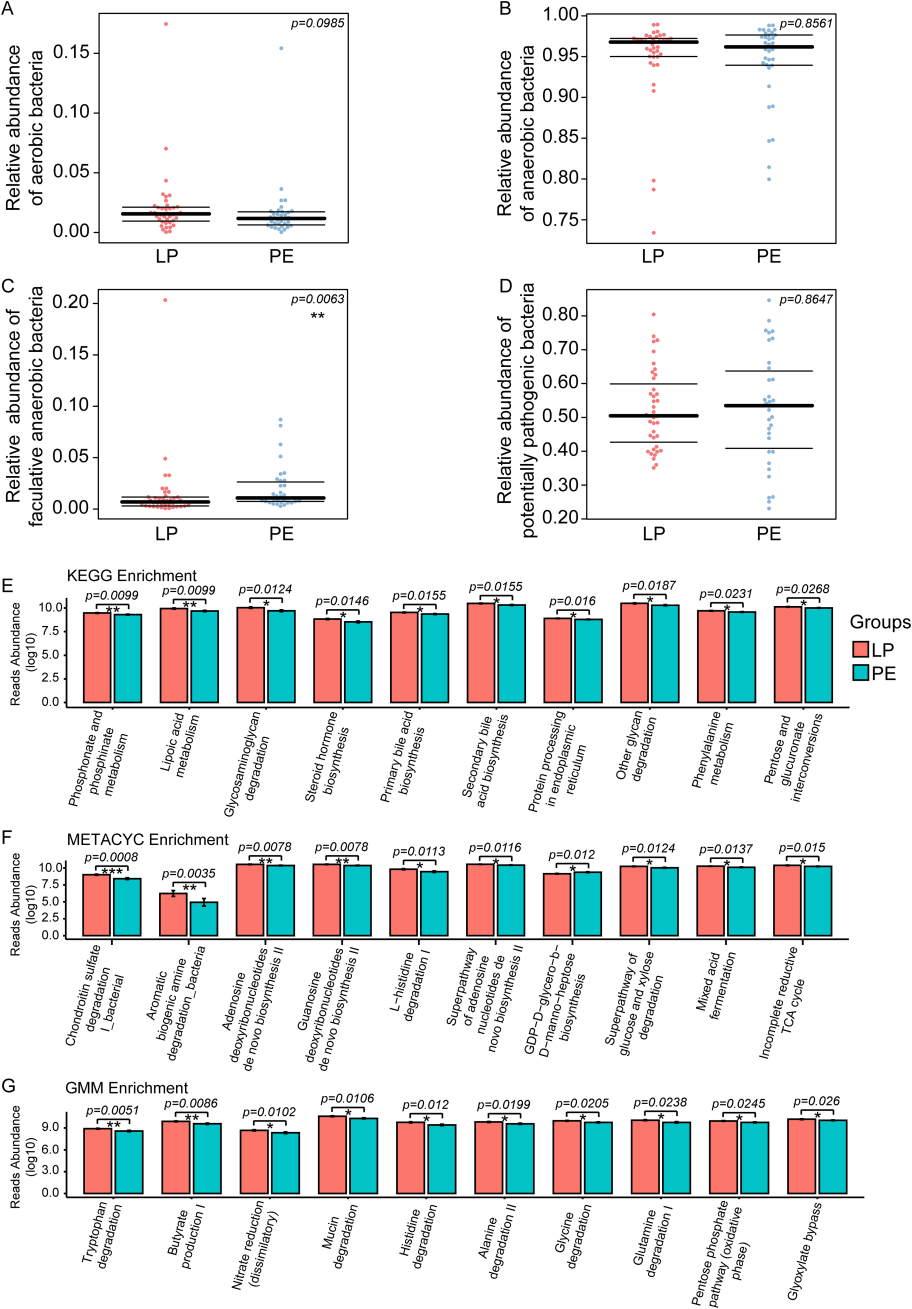
Bugbase Analysis was used to investigate the phenotypic characteristics of intestinal bacteria between the LP group and the PE group, including aerobic bacteria, anaerobic bacteria, facultative anaerobic bacteria, and potentially pathogenic bacteria. No significant differences in the relative abundance of aerobic bacteria (p=0.0985) **(A)**, anaerobic bacteria (p=0.8561) **(B)**, and potentially pathogenic bacteria (p=0.8647) **(D)** except for facultative anaerobic bacteria (p=0.0063) **(C)** were observed between these two groups. Picrust2 functional predictive analysis was employed to predict differences in KEGG, METACYC, and GMM modules between the LP and PE groups. The top 10 differential signaling pathways in KEGG **(E)**, METACYC **(F)**, and GMM **(G)** between these two groups. *p<0.05, **p<0.01, ***p<0.001.

To investigate the phenotypic characteristics of gut bacteria in patients with PE, aerobic bacteria, anaerobic bacteria, facultative anaerobic bacteria, and potentially pathogenic bacteria were enriched by Bugbase Analysis between the LP group and the PE group. We observed no significant differences in the relative abundance of aerobic bacteria (p=0.0985), anaerobic bacteria (p=0.8561), and potentially pathogenic bacteria (p=0.8647) except for facultative anaerobic bacteria (p=0.0063) between these two groups (**Figures 3A–D**). Meanwhile, Picrust2 Analysis was employed to predict differences in KEGG, METACYC, and GMM modules between the LP and PE groups, and the corresponding top 10 significant differences were shown in **Figures 3E–G**. Noteworthy, multiple KEGG signaling pathways related to organic compound metabolism were abnormal in the PE group, such as lipoic acid metabolism (p=0.0099), glycosaminoglycan degradation (p=0.0124), steroid hormone biosynthesis (p=0.0146), and primary bile acid biosynthesis (p=0.0155) (**Figure 3E**).

### Differences in plasma levels of AEA and serum levels of VitD between the LP group and the PE group

**Figure 4:**
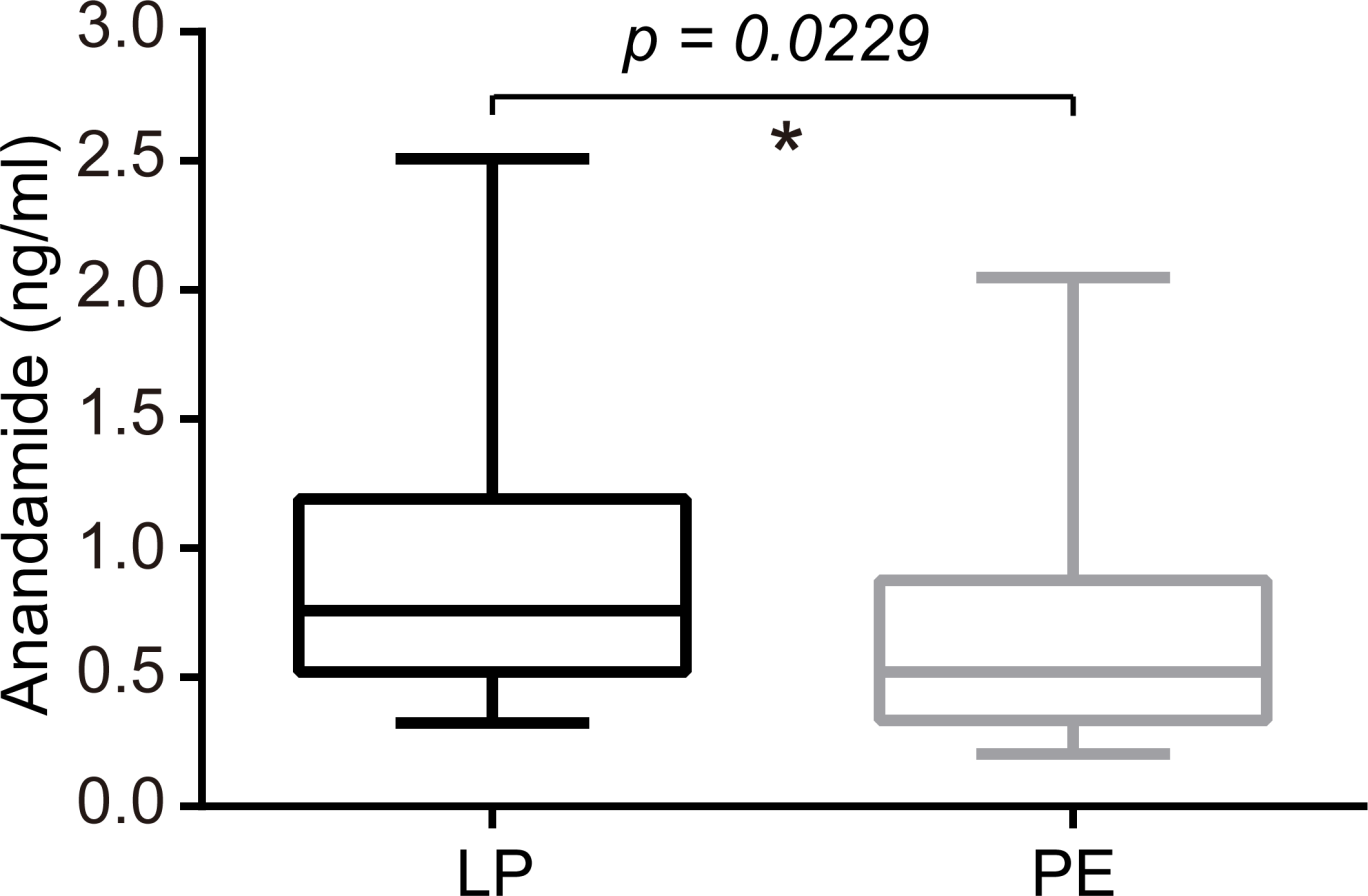
Serum levels of AEA were significantly lower in the PE group than in the LP group (P=0.0229). The median concentrations with interquartile ranges (IQR) were 0.760 (0.521-1.190) ng/ml for the LP group and 0.522 (0.334-0.8775) ng/mL for the PE group. Statistical analysis was performed by the Mann-Whitney test. Middle line: median; box: interquartile range (25-75 percentile). *p<0.05.

**Figure 5:**
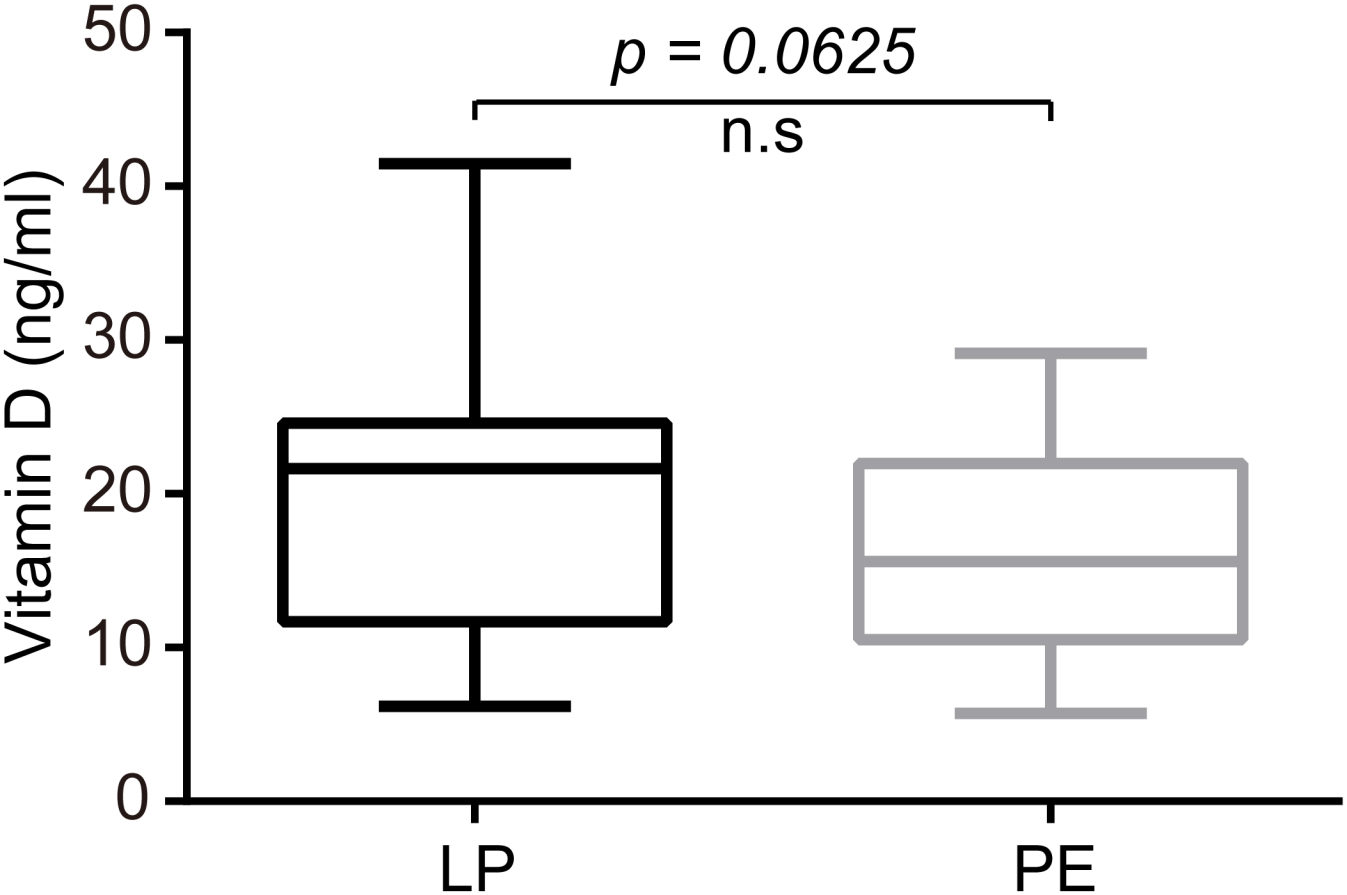
Patients with PE exhibited markedly lower serum concentrations of VitD compared to healthy pregnant women (P=0.0625). The median concentrations with interquartile ranges (IQR) were 21.66 (11.67–24.60) ng/mL for the LP group and 15.61 (10.51–22.00) ng/mL for the PE group. Statistical analysis was performed by the Mann-Whitney test. Middle line: median; box: interquartile range (25-75 percentile).

Plasma levels of AEA were significantly lower in the PE group than in the LP group (P<0.05), whose median concentrations with interquartile ranges (IQR) were as follows: 0.522 (0.334-0.8775) ng/mL versus 0.760 (0.521-1.190) ng/mL (**Figure 4**). Meanwhile, preeclamptic patients exhibited markedly lower serum concentrations of VitD compared to healthy pregnant women (15.61 (10.51–22.00) ng/mL for the PE group vs. 21.66 (11.67–24.60) ng/mL for the LP group, p=0.0625; **Figure 5**).

### Correlations analysis among PE, 10 demographic characteristics, plasma levels of AEA, serum levels of VitD, top 6 marker genera, and fetal birth weight

**Figure 6:**
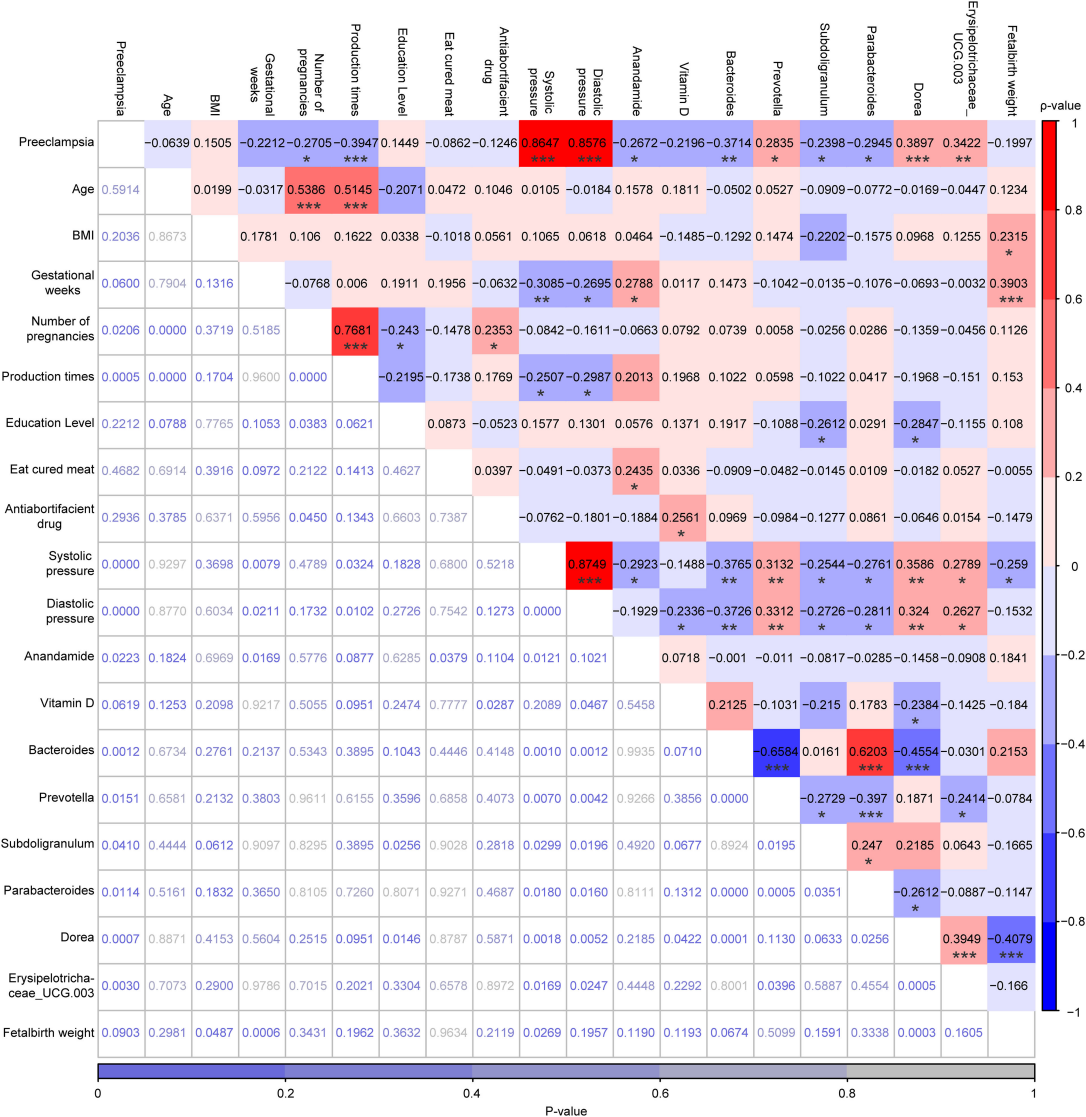
Correlation analysis among PE status, 10 demographic characteristics, plasma levels of AEA, serum levels of VitD, and the relative abundances of the top 6 marker genera for the LP and PE groups, which was performed by Spearman’s Rank Correlation Analysis. The values highlighted in various colors were the correlation coefficients and their scale was on the right. The values at the bottom that were not highlighted were the P-values and their scale was at the bottom. *p < 0.05, **p < 0.01, and ***p < 0.001.

As shown in **Figure 6**, PE was significantly positively correlated with systolic pressure (ρ=0.86, *P*<0.001), diastolic pressure (ρ=0.86, *P*<0.001), prevotella (ρ=0.28, *P*<0.05), *Dorea* (ρ=0.39, *P*<0.001), and *Erysipelotrichaceae_UCG-003* (ρ=0.34, *P*<0.01), whereas was significantly negatively correlated with number of pregnancies (ρ=-0.27, *P*<0.05), production times (ρ=-0.39, *P*<0.001), plasma levels of AEA (ρ=-0.27, *P*<0.05), *Bacteroides* (ρ=-0.37, *P*<0.01), *Subdoligranulum* (ρ=-0.24, *P*<0.05), and *Parabacteroides* (ρ=-0.29, *P*<0.05). Although both plasma levels of AEA and serum levels of VitD were lower in the PE group than in the LP group, no significant correlation was observed between these two risk factors (ρ=0.07, *P*>0.05). Moreover, AEA was not significantly correlated with any of the top 6 marker genera, whereas only VitD was significantly negatively correlated with *Dorea* (ρ=-0.24, *P*<0.05). Interestingly, significant correlations were also identified among most of the top 6 marker genera, such as between *Bacteroides* and *Prevotella* (ρ=-0.6584, *P*<0.001), between *Bacteroides* and *Parabacteroides* (ρ=0.6203, *P*< 0.001), between *Bacteroides* and *Dorea* (ρ=-0.4554, *P*<0.001), among others (**Figure 6**).

## Discussion

PE is a complex pregnancy disorder involving hypertension and dysfunction across multiple systems including placental, vascular, renal, and immune systems. It can be classified with or without severe features, and delivery remains the definitive treatment (Robillard et al., 2017). Despite numerous studies highlighting the potential roles of intestinal bacteria, AEA, and VitD, none have established them as reliable biomarkers for predicting the onset of PE. Moreover, their interactions in late-stage pregnancy women remain poorly understood. In our study, α diversity did not significantly differ between the LP and PE groups, whereas β diversity showed notable distinctions. *Subdoligranulum, Parabacteroides*, *Bacteroides* had suggestive protective effects against PE, while *Prevotella*, *Erysipelotrichaceae_UCG-003*, and *Dorea* might induce the disease occurrence. Furthermore, women with PE had a significantly lower plasma level in AEA and a marked decrease in serum VitD compared to normal late-pregnant women. Lastly, although the plasma level of AEA was not significantly correlated with VitD or any of the top 6 marker genera, VitD was significantly negatively correlated with the relative abundance of *Dorea*, a novel finding in this context.

The human gut microbiome exhibited significant variability among individuals, and microbiomes of both healthy and diseased individuals often show overlapping patterns with some degree of differentiation when analyzed using unsupervised learning methods (He et al., 2018). However, our study revealed significant differences in β diversity between the LP group and the PE group, indicating significant differences in the composition of gut microbiota in patients with PE. Of particular interest were the dysbiosis patterns showing significant depletion of *Subdoligranulum*, *Parabacteroides*, and *Bacteroides* in the PE group. Consistent with previous findings, patients with PE exhibited lower abundances of *Subdoligranulum* compared to healthy pregnant controls (Chang et al., 2020). Interestingly, the decreased level of *Subdoligranulum* observed in patients with PE was butyric acid-producing gut genus (Louis et al., 2010; Riviere et al., 2016; Qin et al., 2019), suggesting that the reduction in butyric acid in these individuals might be attributed to the deficiency of this gut bacterium. Several studies have reported that the levels of butyrate are lower in PE cases than in controls (Chang et al., 2020; Altemani et al., 2021; Jin et al., 2022). The protective role of butyrate against PE was supported by several biological mechanisms. For instance, animal studies indicated that elevating butyrate levels in the colon significantly reduced blood pressure, potentially by acting on the vagus nerve and GPR41/43 receptors (Onyszkiewicz et al., 2019; Yang et al., 2019), which might help counteract hypertension, a key risk factor for PE. In addition, butyrate was known to modulate the immune response, enhance gut barrier integrity, and support healthy placental development (Hu et al., 2019; Zietek et al., 2021). Furthermore, higher butyrate levels were linked to decreased circulating endotoxins and inflammatory cytokines, both of which were implicated in PE (Zietek et al., 2021). Given the influence of dietary interventions on butyrate, it presented a promising target for preventing PE (Frederick et al., 2005; Qiu et al., 2008).

As anaerobic Gram-negative bacilli, both *Parabacteroides* and *Bacteroides* are *Bacteroidaceae* members, and constitute a substantial proportion of gut microbiota. Consistent with previous findings, patients with PE exhibited lower abundances of *Parabacteroides* and *Bacteroides* compared to healthy pregnant controls (Lv et al., 2022; Zhao et al., 2022; Lv et al., 2024), suggesting their protective roles in the occurrence of PE.

Cekanaviciute et al. reported that *Parabacteroides distasonis* stimulated the expression of anti-inflammatory IL-10 in human CD4^+^CD25^+^T cells and IL-10^+^FoxP3^+^Tregs mouse models (Cekanaviciute et al., 2017). Although limited directed associations have been reported between PE and *Parabacteroides*, patients with PE exhibited an enhanced inflammatory response accompanied by decreased blood levels of IL-10 in the third trimester of pregnancy in comparison to controls (Sahin et al., 2015; Aggarwal et al., 2019), indicating that *Parabacteroides* might contribute to the progression of PE by regulating immunity. Meanwhile, some *Parabacteroides* species could also secret SCFAs, including acetate, propionate, and butyrate (Ahmed et al., 2019; Fu et al., 2019). Except for the pervasive protective effects of butyrate in PE cases, the concentrations of acetate and propionate in PE cases were also significantly lower than in controls (Bock, 1994; Bahado-Singh et al., 2012, 2015; Jin et al., 2022). In addition, many studies have reported the crucial roles of *Bacteroides species* in immune and metabolic processes (Wexler, 2007; Lv et al., 2016; Zafar and Saier, 2021). *Bacteroides* are significant contributors to the biosynthesis of lipopolysaccharides (LPS), which can induce inflammation during pregnancy. However, recent studies and our findings indicated significantly lower relative abundances of *Bacteroides* in the PE group compared to the LP group (Lv et al., 2022; Zhao et al., 2022). Evidence of different beneficial functions from various *Bacteroides* strains may partly explain this phenomenon. *Bacteroides vulgatus* and *Bacteroides dorei* are the predominant species within the *Bacteroides* genus in the human gut microbiome, and their LPS compounds penta- and tetra-acylated lipid A differ structurally from the hexa-acylated LPS found in Escherichia coli (Vatanen et al., 2016). what’s more, the LPS compounds penta- and tetra-acylated lipid A of these two *Bacteroides* species could elicit reduced Toll-like receptor 4 (TLR4) responses (Vatanen et al., 2016). The activation and maturation of dendritic cells via TLR signaling promote the upregulation of major histocompatibility complex (MHC) molecules, costimulatory factors, and cytokine production, ultimately leading to T-cell activation (Ardavin et al., 2004; Mills, 2011). In addition, *Bacteroides fragilis* is also a well-studied representative known for its immune-regulatory abilities. *Bacteroides fragilis* ATCC25285 has been shown to protect against intestinal inflammatory diseases caused by pathogenic *Helicobacter hepaticus* (Mazmanian et al., 2008). Another strain, *Bacteroides fragilis* ZY-312, facilitates the polarization of bone marrow-derived macrophages towards the M1 phenotype, promoting the phagocytosis of pathogens (Deng et al., 2016). Interestingly, Wang et al. also observed differences in the relative abundances of *Bacteroides* species between patients with PE and healthy controls, with lower levels of *Bacteroides stercoris* and higher levels of *Bacteroides coprocola* and *Bacteroides fragilis* in patients with PE compared to healthy controls (Wang et al., 2019). Taken together, given the diverse roles of *Bacteroides* species in immunomodulation, transitioning from 16S rRNA sequencing to metagenomics sequencing would be essential for comprehensive understanding in future studies.

Meanwhile, the relative abundances of *Prevotella* and *Erysipelotrichaceae_UCG-003* in the PE group were significantly higher than in the LP group, indicating these bacteria had abilities to induce the occurrence of PE in this study. Inflammation of the chorionic plate is linked to the presence of several bacterial species in the preeclamptic placenta, including *Prevotella*, *Bacillus cereus*, *Listeria*, *Salmonella*, *Escherichia*, *Klebsiella pneumonia*, *Anoxybacillus*, *Variovorax*, *Porphyromonas*, and *Dialister* (Amarasekara et al., 2015). Moreover, women with severe PE exhibited a higher relative abundance of *Prevotella bivia* in their vaginal microbiota, along with elevated plasma levels of the pro-inflammatory cytokine TNF-α, compared to healthy controls (Hung et al., 2004; Lin et al., 2020). Although women with gestational diabetes mellitus exhibited a higher abundance of genus *Erysipelotrichaceae UCG-003* in comparison with normoglycemic women (Ferrocino et al., 2018; Vavreckova et al., 2022), research about the effects of *Erysipelotrichaceae_UCG-003* on PE is still limited. Enhanced relative abundances of *Erysipelotrichaceae* in patients with colorectal cancer, and animal models of 1, 2-dimethylhydrazine-induced colon cancer or inflammatory bowel diseases, have been reported (Chen et al., 2012; Zhu et al., 2014; Schaubeck et al., 2016), indicating its importance in inflammation-related disorders.

The composition of gut microbiota can be influenced by lifestyle factors, such as VitD intake. VitD deficiency has been associated with dysbiosis of gut microbiota and gastrointestinal inflammation, with studies showing that VitD supplementation can significantly enhance gut microbial diversity and increase the ratio of *Bacteroidetes* to *Firmicutes* (Singh et al., 2020). Additionally, VitD intake has also been linked to higher abundances of *Akkermansia*, *Bifidobacterium*, *Coprococcus*, and *Lactococcus*, while reducing the abundances of *Porphyromonas*, *Ruminococcus*, *Veillonella*, and *Erysipelotrichaceae* (Kanhere et al., 2018; Naderpoor et al., 2019; Charoenngam et al., 2020; Singh et al., 2020). In this study, although there was no significant reduction in the serum levels of VitD among women with PE compared to normal late-pregnant women, a significant negative correlation was identified between the VitD levels and the relative abundance of *Dorea*. Consistent with findings from a randomized controlled trial in men with pre-diabetes and VitD deficiency, VitD supplementation could decrease the relative abundance of several genera within the *Lachnospiraceae* family, including *Dorea*, *Blautia*, *Roseburia*, and *Ruminococcus* (Ciubotaru et al., 2015). Meanwhile, in an observational cohort study, alcohol-dependent subjects with increased intestinal permeability exhibited higher relative abundances of *Dorea*, indicating the important role of *Dorea* in the maintenance of gut barrier integrity (Leclercq et al., 2014). In addition, we also inferred the mechanism underlying how VitD might influence the gut microbiota in patients with PE. VitD contributed to the maintenance of mucosal barrier integrity by enhancing the expression of tight junction and adherent junction proteins and inhibiting epithelial cell apoptosis, thereby preserving gut barrier integrity and function (Wu et al., 2010; Ooi et al., 2012; Clark and Mach, 2016). The disruption of gut barrier function could lead to increased susceptibility to pathogenic infections and heightened inflammation, which, in turn, adversely affects the gut microbiota (Jin et al., 2015; Waterhouse et al., 2019; Li et al., 2023). Moreover, the disruption of gut barrier integrity could also result in the colonization of gut bacteria in the uterine cavity, thus causing PE (Chen et al., 2020). Meanwhile, Vitamin D receptors (VDRs), which are abundantly expressed in intestinal enterocytes, especially in the proximal colon, facilitate the production of antimicrobial peptides like cathelicidins, defensins, claudins, and zonulin occludens (Wu et al., 2010; Ooi et al., 2012; Clark and Mach, 2016). This selective elimination of pathogenic bacteria provides a greater chance for beneficial bacteria to colonize. As a whole, VitD status can influence gut microbiota composition by promoting anti-inflammatory responses and reducing infection risk, making it a potentially modifiable factor in preventing PE by maintaining the balance of gut microbiota (Talsness et al., 2017).

There were several limitations in this study. Firstly, the sample size was relatively small and collected from a single center. Future research with larger sample sizes and data collected from multiple centers will be necessary to validate our findings. Secondly, dietary and nutrient intake information was gathered solely through questionnaires, which may not provide precise assessments. Thirdly, our study primarily observed dysbiosis in gut microbiota among late-pregnant women with PE without delving into the underlying mechanisms. Lastly, we lacked blood and fecal samples from patients with severe PE, as it was challenging to recruit individuals matching the age, gestational age, and BMI criteria of normal late-pregnant women or those with PE. Currently, we are collecting samples from normal late-pregnant women, patients with PE, and severe PE to further substantiate our preliminary findings.

## Conclusions

In summary, the profile of gut microbiota differed notably between the PE and LP groups. Gut bacteria involved in regulating immune response and gut barrier integrity (e.g. *Subdoligranulum*, *Bacteroides*, and *Dorea*), showed significant alterations in PE patients, suggesting their potential roles in the onset of PE. Moreover, while the plasma levels of AEA were not significantly correlated with the serum levels of VitD or any of the 6 identified marker genera, there was a significant negative correlation between VitD and *Dorea*, indicating VitD and gut microbiota have the potential to be combined targets for early diagnosis and management of PE.

## Data Availability

The datasets presented in this study can be found in online repositories. The names of the repository/repositories and accession number(s) can be found in the article/**Supplementary Material**.
