# Supplementary material for: Gut microbiota interacting with vitamin D but not anandamide might contribute to the pathogenesis of preeclampsia: a preliminary study
Source: Front Cell Infect Microbiol. 2025 Feb 5;14:1469054. doi: 10.3389/fcimb.2024.1469054 (PMC11835824; doi:10.3389/fcimb.2024.1469054)
Supplement: Supplementary file 1 [file DataSheet1.pdf]

Gut microbiota interacting with vitamin D but not anandamide might contribute to the pathogenesis of preeclampsia

Xiao-Qiang Han[1,2†], Hui-Hui Jiang[3†], Meng-Ling Chen[1,2†], De-Yang Han[1,2], Su-Fen Zhou[2,4], Jin-Wen Wang[3], Shu-Shen Ji[3], Ling-Yun Wang[3], Jing-Wei Lou[3*], Ming-Qun Li[1,2*]

[1]Department of obstetrics and gynaecology, Xiangyang No. 1 People's Hospital, Hubei University of Medicine, Xiangyang, China

[2]Hubei provinical clinical research center for accurate fetus malformation diagnosis, Xiangyang, China

[3]Zhangjiang Center for Translational Medicine, Shanghai Biotecan Pharmaceuticals Co., Ltd., Shanghai, China

[4]Department of Ultrasound, Xiangyang No. 1 People's Hospital, Hubei University of Medicine, Xiangyang, China

*CORRESPONDENCE

Jing-Wei Lou

jingweilou@biotecan.com

Ming-Qun Li

essay198182@163.com

†These authors contributed equally to this work and share first authorship.

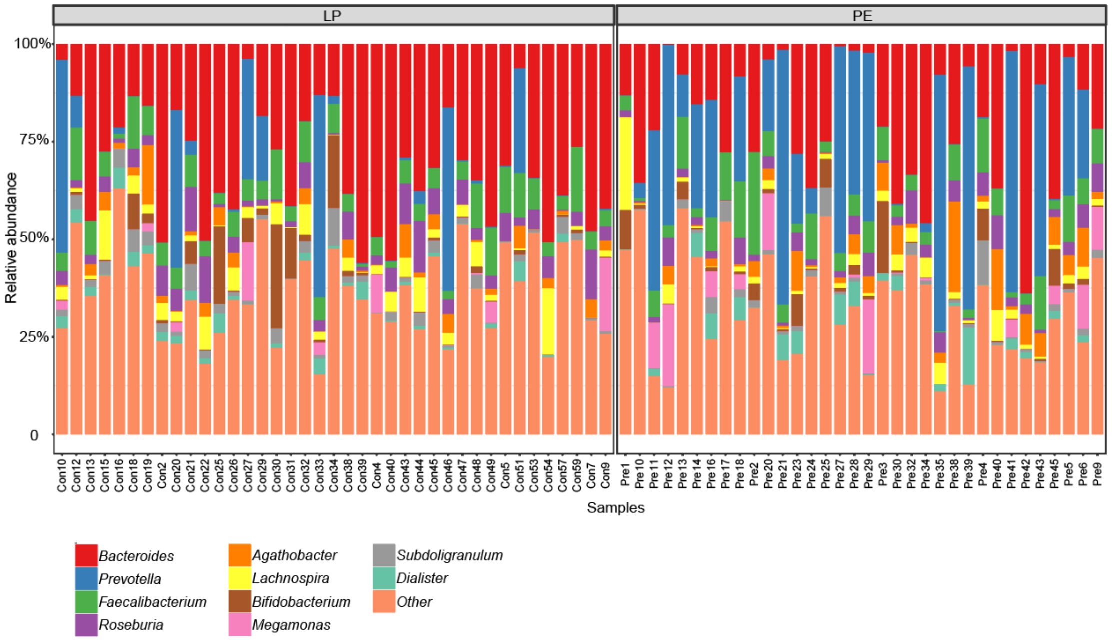

Supplementary Figure 1. Composition and distribution of gut microbiota at the generic level in healthy late-pregnant women (LP, n=39) and preeclamptic patients (PE, n=34). The top 10 shared genera with high relative abundance were depicted in different colors, while the less abundant genera were grouped as 'other.'
